# External tissue expansion to salvage failed scalp and forehead reconstruction: a case report

**DOI:** 10.1080/23320885.2022.2123809

**Published:** 2022-09-14

**Authors:** Peter Y. W. Chan, Elina Patel, Ethan Paulin, Ajul Shah

**Affiliations:** The Institute for Advanced Reconstruction, Shrewsbury, NJ, United States of America

**Keywords:** Tissue expansion, scalp reconstruction, cranial reconstruction, salvage reconstruction, salvage

## Abstract

The reconstruction of forehead and scalp defects is a difficult task. Common reconstructive methods are associated with multiple complications and may fail, requiring a difficult second surgery. We present the use of external tissue expansion as a method to achieve effective closure of a failed scalp and forehead reconstruction.

## Introduction

The reconstruction of forehead and scalp defects is a difficult task for plastic and reconstructive surgeons [1]. Common methods to close scalp and forehead defects include tissue transfer-based reconstruction such as skin grafts or flaps [2,3]. However, these methods come with a variety of aesthetic and functional complications, including the inability to ideally match tissue in the recipient area [4]. Additionally, graft or flap failure is a concerning possible complication [3,5,6]. In the case of primary reconstruction failure, a secondary reconstructive technique must be undertaken. However, these techniques are often more complicated and difficult than the original, and functional and aesthetic outcomes may be compromised [7–10]. As such, there is a need for simpler, more efficacious methods to salvage failed reconstruction in the scalp and forehead.

Tissue expansion is a reconstructive technique where continuous force is applied to wound edges, inducing tissue proliferation and expansion. Its use in primary scalp and forehead reconstruction has been reported [11,12]. Herein, we present a case demonstrating a novel use of external tissue expansion *via* a modern expander (DermaClose, Synovis). Specifically, external tissue expansion was used as a secondary reconstructive method to salvage failed primary scalp and forehead reconstruction. This technique can achieve excellent functional and cosmetic outcomes by utilizing native tissue for closure while avoiding many of the complications associated with skin graft and flap reconstruction.

## Case report

**Figure 1.**
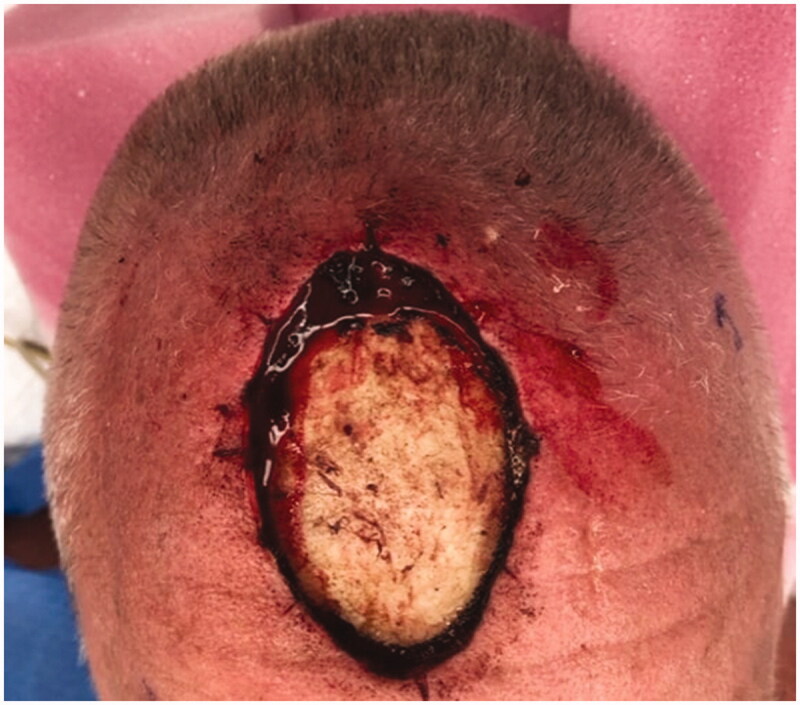
A 65-year-old male presented with basal cell carcinoma. Oncologic resection produced a 48 cm^2^ (8 cm × 6 cm) wound with exposed calvarium without periosteum.

A 65-year-old male presented to the authors with basal cell carcinoma on the forehead. The patient underwent resection of the affected area, which produced a large 48 cm^2^ (8 cm × 6 cm) wound (Figure 1). Specifically, Mohs surgery was performed with a permanent pathology report delineating clear margins. The wound had exposed calvarium without periosteum, which was removed during the cancer resection. The patient underwent reconstruction with H-type flap advancements with a superior incision along the hair line, which were notably tight but produced primary closure after flap transfer.

**Figure 2.**
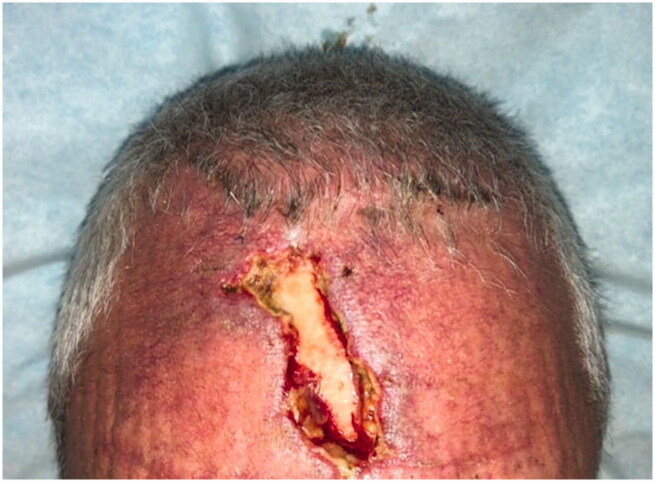
The patient underwent H-type flap advancements with a superior incision along the hairline. At the 2-week follow-up, the patient demonstrated partial flap necrosis due to a lack of tissue and excess tension.

At 2 weeks post-operative follow-up, the patient demonstrated partial flap necrosis (Figure 2). Specifically, a 7.5 cm^2^ (1.5 cm × 5 cm) defect remained. Options were discussed with the patient with concern that burring of the bone would be necessary for skin graft reconstruction to be successful, which would leave the patient with severe unaesthetic reconstruction. Instead, the authors utilized external tissue expansion *via* 1 DermaClose device.

**Figure 3.**
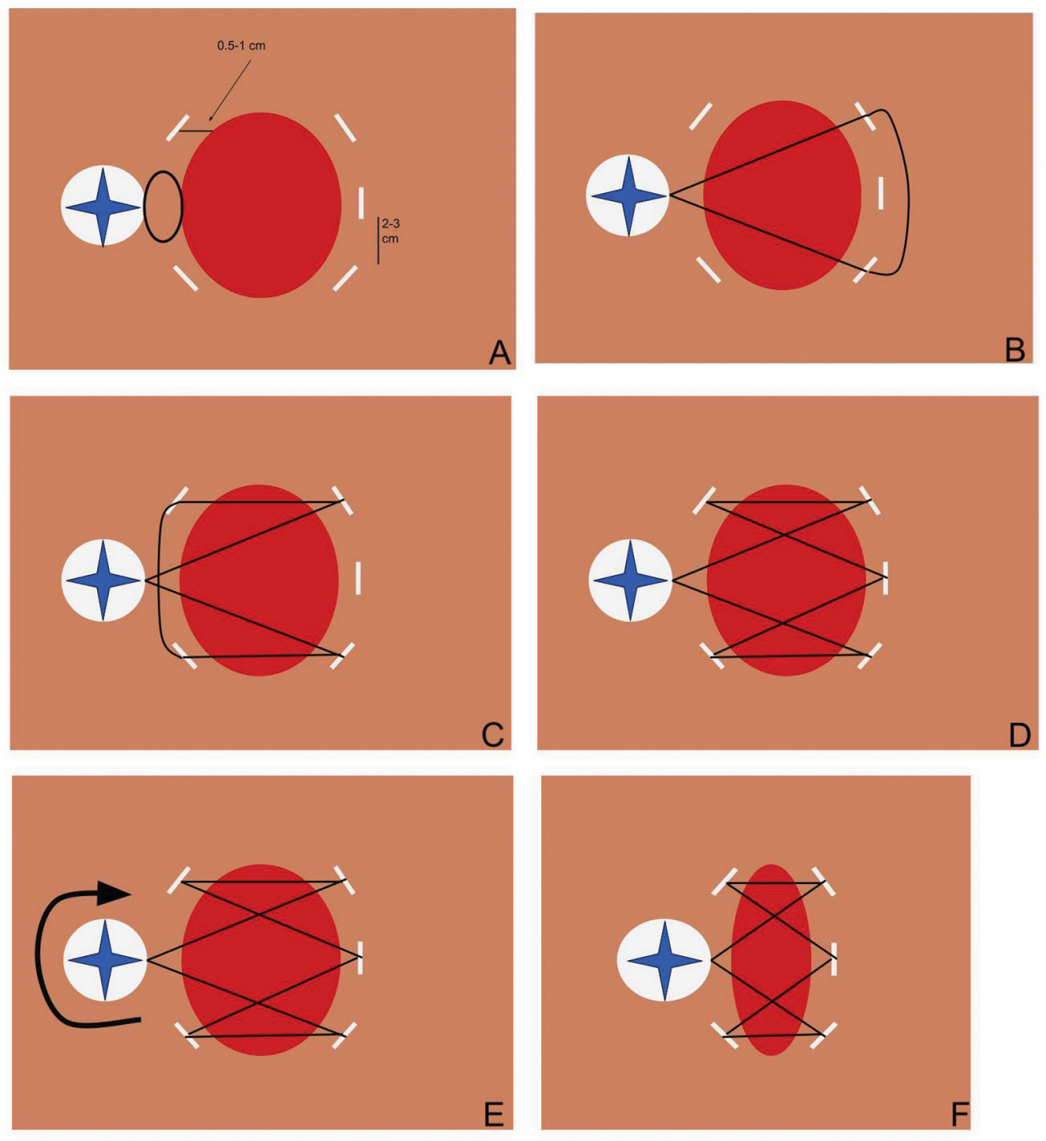
A diagram demonstrating the appropriate application of the DermaClose device.

**Figure 4.**
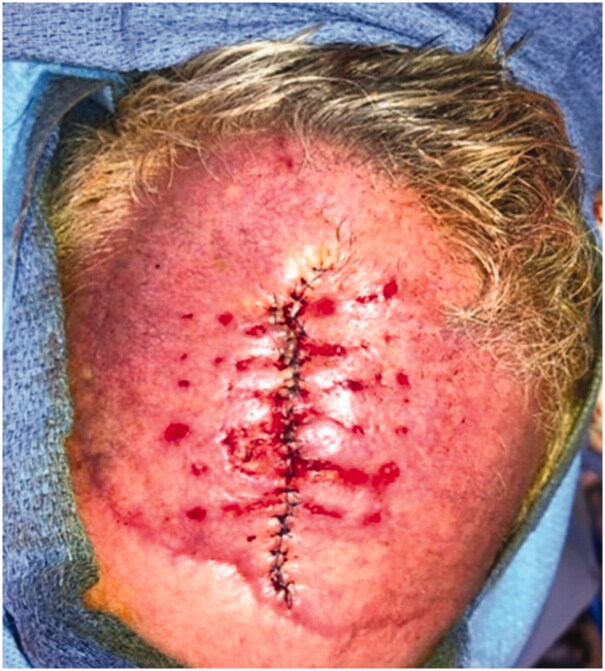
Delayed primary closure was achieved after external tissue expansion for 7 days.

**Figure 5.**
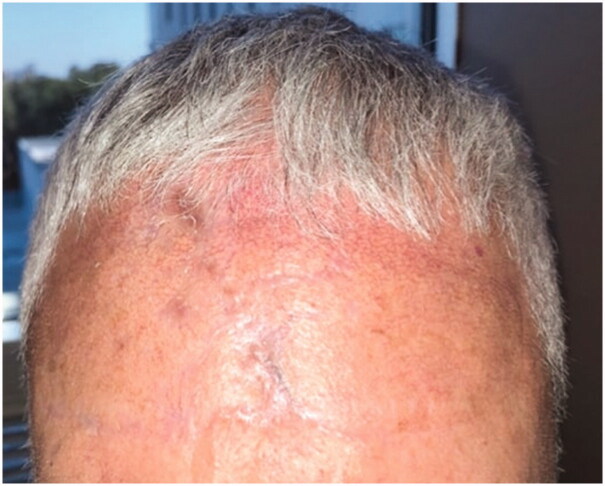
At the 12-week follow-up, the patient demonstrated excellent aesthetic results with minimal scarring.

The wound was thoroughly cleaned and debrided. The wound edges were undermined by one-half the width of the wound. Skin anchors were affixed to the skin with staples, 2–3 cm from each other and 0.5–1 cm from the wound edge. The tension line was laced around the skin anchors in a “VMW” pattern (Figure 3). The tension controller was wound clockwise until an audible click was heard, then locked by pressing on the controller. The device was left until sufficient tissue was expanded for closure. In our case, this was 7 days. The range of time needed for tissue expansion is dependent on the size and location of the wound as well as the patient’s skin properties. However, it is typically no longer than 7–11 days. After 7 days, tissue expansion allowed for delayed primary closure (Figure 4). At a 12-week post-closure follow-up, the patient demonstrated excellent aesthetic results with minimal scarring and full sensation in the area (Figure 5).

## Discussion

After the failure of the primary reconstructive technique in the forehead and scalp, surgeons are faced with a difficult task. Skin grafting reconstruction may be considered. However, a skin graft requires a vascular bed, and scalp and forehead defects are often denuded without periosteum [9]. Although the bone can be burred to create a foundation of vascularized tissue, the healing process is lengthy and requires regular dressing changes [9]. Additionally, skin grafting in the scalp and forehead is often associated with poor cosmetic results, often leaving a white, patchy appearance [8]. A second tissue-transfer-based reconstruction may be considered; however, these secondary procedures are often even more challenging than the primary technique due to a combination of the first-choice flap already being used, a scarcity of existing vessels to create or receive a new flap, and surgical scarring in the area [5,7,10]. The complication rate of all secondary flap procedures in head and neck reconstruction after initial flap failure has been reported as high as 21–22% [7,13]. Surgeons may also be hesitant to undertake a secondary tissue-transfer-based reconstruction when there was no clear cause of failure in the first.

We present external tissue expansion *via* a modern expander, the DermaClose, as a safe, effective, and simple method to salvage failed reconstruction in the head and scalp. Since its first use in 1976, tissue expansion has grown in popularity as a method to close soft tissue defects [14]. In tissue expansion, a device subjects the tissue adjacent to a wound to a constant mechanical force; as a result, the skin both stretches and proliferates into additional tissue which can be used for delayed primary closure [14]. Tissue expansion consists of both internal and external methods depending upon the location of the device inducing tissue proliferation.

There are many benefits of tissue expansion, including both internal and external methods, over other reconstructive techniques. Placement and removal of tissue expanders are simple and can be done under local or regional anesthesia, avoiding the perioperative risks of longer procedures under general anesthesia such as skin grafting or flap reconstruction [14]. Tissue expansion has been associated with lower complication rates compared with tissue transfer-based techniques [14]. Additionally, tissue expansion creates native tissue for closure, allowing for reconstruction with full-thickness, sensate, and like-for-like tissue. This avoids many of the potential complications of tissue transfer-based reconstruction, including the creation of a donor site, aesthetic mismatch to the recipient area, and sensation-related issues.

Tissue expansion as a method to close defects in the head and scalp has been reported [11,12]. Older literature reports the use of internal tissue expansion, which requires the implantation of a silicone balloon beneath the skin adjacent to the defect that is progressively filled with saline to stretch the overlying tissue [11,12]. However, internal tissue expansion has been associated with higher rates of complication compared with external expansion [14,15]. Additionally, internal expanders have poor cosmetic appearance during the expansion process, which is problematic for some patients [15]. As such, many surgeons have shifted to external tissue expansion to successfully close primary forehead and scalp defects [1,16,17].

Our case is unique from the existing literature because, as far as the authors are aware, external tissue expansion has never been described as a method to salvage failed primary scalp and forehead reconstruction. While there are other salvage methods, they are often complex and may come with a variety of complications [2,7,13]. In comparison, external tissue expansion is simple and carries the same benefits of the overall technique, including producing like-for-like tissue in a cosmetically important area, avoiding lengthier procedures with higher perioperative risk profiles, and having lower complication rates.

External tissue expansion is a powerful tool but requires appropriate patient selection. The expansion process may be associated with discomfort or pain and patients should be made aware of this possibility. The expander’s skin barbs may be problematic for patients with thin or fragile skin. In general, this can be ameliorated by anchoring the device further from the wound and placing a dressing underneath the anchors. However, excessively fragile skin may be a contraindication. Areas of the body with limited pliable tissue are not as amenable to tissue expansion.

In our case, the patient underwent resection of basal cell carcinoma on the forehead, producing a relatively large defect. H-type flap advancements were utilized to close the defect. However, the flap failed due to a paucity of tissue and excessive tension. Conventionally, the patient’s outlook would be grim. Potential treatments would include a skin graft with the need for bone burring and lengthy recovery or a secondary flap with its associated complications, difficulty, and risk. However, we were able to achieve successful, simple closure *via* external tissue expansion without a lengthy or complicated procedure. The patient demonstrated excellent aesthetic results, minimal scarring, and intact sensation in the area of reconstruction.

In conclusion, our case shows that external tissue expansion demonstrates excellent potential as a method to salvage failed reconstruction in the head and scalp. Further studies with larger sample sizes are needed to assess the efficacy of external tissue expansion with other types of head/neck wounds.

## Ethical approval

The study was performed in accordance with the principles of the Declaration of Helsinki.

## Statement of informed consent

Informed consent was obtained from the participants included in the study.
